# Inappropriate laboratory testing in internal medicine inpatients: Prevalence, causes and interventions

**DOI:** 10.1016/j.amsu.2020.02.002

**Published:** 2020-02-07

**Authors:** B.E.L. Vrijsen, C.A. Naaktgeboren, L.M. Vos, W.W. van Solinge, H.A.H. Kaasjager, M.J. ten Berg

**Affiliations:** a University Medical Center Utrecht, Department of Internal Medicine, Utrecht, the Netherlands; b Julius Center for Health Sciences and Primary Care, University Medical Center Utrecht, Utrecht University, Utrecht, the Netherlands; c University Medical Center Utrecht, Laboratory of Clinical Chemistry and Haematology, Utrecht, the Netherlands

**Keywords:** Laboratory medicine, Internal medicine, Overtesting, Overutilization

## Abstract

**Background:**

To reduce overutilization of laboratory testing many interventions have been tried, but selecting the most effective intervention for a given setting is challenging. To be sustainable, interventions need to align with healthcare providers' needs and daily practices. This study aimed to assess the extent of overutilization and the perspectives of healthcare providers, which may be used to guide the choice of intervention.

**Methods:**

The extent of inappropriate laboratory testing in internal medicine inpatients was evaluated using a database. Surveys and focus groups were used to investigate healthcare providers' perceptions on its causes and solutions.

**Results:**

On average, patients had 5.7 laboratory orders done during the first week of admission, whereas guidelines advise performing laboratory testing no more than twice per week. Repeat testing of normal test results occurred in up to 85% of patients. The frequency of laboratory testing was underestimated by survey responders, even though the majority of responders (78%) thought that laboratory tests are ordered too frequently. Residents were considered to be most responsible for laboratory test ordering.

The primary causes of overutilization discussed were personal factors, such as a lack of awareness and knowledge, as well as feelings of insecurity. Regarding possible solutions, residents generally recommended educational interventions, whereas specialists tended to favour technical solutions such as lockouts.

**Conclusion:**

Inappropriate laboratory testing is common in internal medicine. The most important causes are a lack of awareness and knowledge, especially in residents. The intervention most favoured by residents is education, suggesting educational interventions may be most applicable.

## Highlights

• In internal medicine inpatients, laboratory testing occurs on average 5.7 times per week, more than guidelines recommend.
• 59% of C-reactive protein (CRP) repeat tests are performed within the recommended minimum testing interval.
• Health care providers underestimate the extent of overutilization of laboratory testing.
• The most important causes of overutilization are a lack of awareness and knowledge, and feelings of insecurity.
• Specialists and residents have different opinions on what the most effective intervention is to reduce overutilization.

## 1 Introduction

Laboratory testing affects up to 70% of downstream treatment decisions [1]. The overutilization of laboratory tests is common and some estimate that one out of every five tests performed is unnecessary [2]. Inappropriate laboratory test utilization increases the potential for diagnostic errors when these tests give false-positive or false-negative results [3,4]. Additionally, given the large volume of laboratory testing, overutilization leads to substantial costs [5,6].

The relevance of the issue of overutilization of laboratory tests is increasingly being recognised, as evidenced by the development of guidelines and campaigns aimed at reducing inappropriate test utilization. For example, the international Choosing Wisely campaign has encouraged professional societies to issue guidelines recommending targeted, deliberate laboratory testing [7]. However, adherence to guidelines is often poor [8].

A systematic review of the literature revealed that many different interventions to reduce inappropriate testing have been investigated, such as educational methods, changes in the ordering system, audit and feedback methods [9]. While all interventions have been shown to reduce unnecessary laboratory testing initially, evidence on long-term sustainability is lacking. Additionally, which interventions are most (cost)-effective is unclear due to the lack of head-to-head comparisons.

At our institution, several small ad hoc initiatives to reduce overutilization have been undertaken, but these initiatives have not yet lead to any sustainable reduction of laboratory test utilization. Effective implementation of innovations in healthcare requires a systematic approach including an analysis of the target audience and the context they work in [10,11].

So, when making a considered choice of which intervention to implement, information on the local practices and attitudes of health care professionals regarding laboratory testing is required.

In order to effectively implement interventions to increase appropriate laboratory testing, we investigated the current practice of laboratory testing at our department of internal medicine, and what health care professionals think about the causes of the surmised inappropriate test ordering as well as their ideas for potential solutions.

## 2 Methods

This study comprises three parts. Firstly, we performed a database study to investigate the appropriateness of laboratory testing. Secondly, we did a survey and thirdly a series of focus group interviews, both to evaluate healthcare workers’ attitudes and perceptions of the barriers and facilitators of appropriate laboratory testing.

This study was deemed to be exempt from review by the Medical Research Ethics Committee of the University Medical Center Utrecht.

### 2.1 Database study

#### 2.1.1 Setting and patient population

For the database study we used data from the internal medicine department of the University Medical Center Utrecht, a 1042-bed academic teaching hospital with about 28,000 clinical and 15,000 day-care hospitalizations and 334,000 outpatient visits annually.

At our hospital, laboratory tests are generally ordered by residents, who primarily manage the care for admitted patients. They are supervised by specialists daily. There are no restrictions on the laboratory tests the physicians can order. Laboratory tests are ordered through the electronic medical record. All tests have to be ordered individually: no fixed panels, e.g. sets of tests that are always ordered together, are used.

The venipunctures are performed by either specially trained laboratory staff or the nursing staff on the ward.

#### 2.1.2 Data source

We collected data on all laboratory requests for patients who had been hospitalised at the general internal medicine ward between June 2011 and December 2016.

Data were obtained from the Utrecht Patient Oriented Database (UPOD), an infrastructure of relational databases comprising data on patient characteristics, hospital discharge diagnoses, medical procedures, medication orders and laboratory tests for all patients treated at the University Medical Center Utrecht since 2004. The UPOD data acquisition and management is performed in accordance with current regulations concerning privacy and ethics. The structure and content of UPOD have been described in more detail elsewhere [12].

#### 2.1.3 Defining and quantifying inappropriate laboratory testing

Several measures of inappropriate overuse of laboratory testing were determined through plenary discussions among the authors. These were based on recommendations from the Choosing Wisely campaign, such as the Netherlands Association of Internal Medicine's recommendation to perform laboratory tests no more than twice a week in clinically admitted patients [13], and the recommendation of the American Society for Clinical Pathology to perform lipase testing instead of amylase in suspected pancreatitis [14], and recommended minimal testing intervals [15,16]. The evaluated measures are presented in Table 1. All analyses were done in R version 3.1.2.

**Table 1:** Database study results.

Measure	Result	Explanation
Average number of laboratory orders per patient per week	Week 1: 5.7	Per patient the number of lab orders were counted per week of admittance. Lengths of admittance was rounded up to the next full week.
	Week 2: 3.2	
	Week 3: 3.2	
	Week 4: 3.9	
Repetition of normal test results	Sodium 82% (n = 2833)	The percentage of tests that are repeated when the test result is within the reference range.
	Potassium 85% (n = 3032)	
	Bicarbonate 83% (n = 597)	
	Creatinin 75% (n = 2142)	
	Leucocytes 71% (n = 2463)	
	GGT 23% (n = 1513)	
	ALP 31% (n = 2530)	
	ALT 33% (n = 2919)	
	AST 34% (n = 2476)	
	LDH 35% (n = 2326)	
	CRP 45% (n = 1257)	
Time from admission to repetition of CRP (N = 923)	167 (18%) within 12 h	For all patients in whom a CRP is tested in the Emergency Department and in whom a repeat CRP test was performed during hospitalization, the time in hours between the CRP testing in the Emergency Department and the first subsequent CRP testing during admission.
	381 (41%) 12–24 h	
	211 (23%) 24–48 h	
	164 (18%) more than 48 h	
Percentage of repeated CRP measurements that led to changes in patient management (N = 509)	6.9% start antibiotics	The fraction of repeat CRP tests that led to initiating, discontinuing or changing antibiotic therapy (defined as a new medication order or a stopping order within 4 h of the CRP test).
	5.1% stop antibiotics	
	0.2% switch antibiotics	
	87.8% no effect on antibiotic treatment	
Inappropriate fixed combinations of tests	Sodium + potassium 95%	E.g. the fraction of lab orders with a sodium test that also include a potassium test
	ALT + AST 97%	
	lipase + amylase 85%	
	CRP and procalcitonin 8%	
	Creatinine + BUN 74%	

A lab order is defined as a single blood collection and can contain one or several individual laboratory tests.

Abbreviations: GGT = Gamma glutamyltransferase, ALP = Alkaline phosphatase, ALT = Alanine transaminase, AST = Aspartate transaminase, LDH = Lactate dehydrogenase, CRP = C-reactive protein, BUN = Blood Urea Nitrogen.

### 2.2 Survey

The survey (Table 2) was developed by consensus through discussions in our team, comprising four topics: perceptions of the frequency of overall laboratory testing, perceptions of who are involved in or responsible for the decision to perform laboratory testing, thoughts on the benefits and harms of laboratory testing, and thoughts on interventions to reduce excessive laboratory testing.

**Table 2:** Survey results.

	Resident	Specialist	Nurse	Overall	
How many times per week are laboratory tests ordered per patient?	2.9	3.8	4.8	3.9	n
On average, how many individual laboratory tests are in one order?	8.2	10.8	6.5	8.2	n
How many times per week are add-ons ordered after laboratory tests have been performed?	1.6	2.0	2.9	2.3	n
What do you think of the amount of laboratory testing that is being ordered?	0.80	0.85	0.71	0.79	% too many or far too many
To what extent do you personally decide which laboratory tests are being performed?	1.00	0.92	0.00	0.55	% most of the time or always
To what extent do you personally decide how often laboratory tests are being performed?	1.00	0.69	0.06	0.50	% most of the time or always
Who is mostly responsible for deciding which laboratory tests are being performed?	0.80	0.69	0.82	0.78	% choosing residents
Who is mostly responsible for the frequency of laboratory testing?	1.00	0.69	0.82	0.83	% choosing residents
To what extent do you agree or disagree with the following statements?					% agree or strongly agree
I can monitor patients better if laboratory tests are performed more often.	0.70	0.18	0.50	0.46	
Daily laboratory testing increases patient safety.	0.10	0.00	0.25	0.14	
On the day of discharge, patients should have laboratory tests performed.	0.00	0.00	0.14	0.05	
If fewer laboratory testing is performed, patient safety will be negatively impacted.	0.20	0.00	0.38	0.21	
If fewer laboratory testing is performed, patient satisfaction will be negatively impacted.	0.13	0.08	0.13	0.11	
I have insight into the costs of laboratory testing.	0.00	0.08	0.06	0.05	
I worry about the costs of laboratory testing.	0.44	0.46	0.21	0.36	
I worry about the negative consequences of laboratory testing for patients.	0.67	0.50	0.47	0.53	
Ordering laboratory tests is a standard topic of discussion during supervision.	0.33	0.69	0.43	0.52	

Invitations to fill out the questionnaire online were sent by e-mail to all nurses, residents and specialists working in the general internal medicine department of the University Medical Center Utrecht.

### 2.3 Focus groups

For the focus group participants a purposive sample was recruited from residents and specialists from the internal medicine department. Potential participants were those who had worked on the ward within the past six months. They were approached face-to-face and none declined to participate. The focus groups were organised between January and May 2018 and were prepared and conducted by three of the investigators (BV, MtB, and CN; an internist working in the same department as the focus group participants, a clinical pathologist and a clinical epidemiologist respectively).

The focus group discussions were set up according to the framework developed by Stalmeijer et al. [17] To encourage an open discussion, residents and consultants were included in separate groups, consisting of five to seven people. Prior to the semi-structured focus groups, a set of questions and topics was prepared based on the results of the database study and the survey. A summary of the survey results was presented to the focus group participants at the start of each meeting.

The number of focus groups was determined by the principle of thematic saturation.

Meetings were scheduled to last for 45–60 min and were held in a staff meeting room after working hours. No other people were present.

Data collection consisted of audio recordings and one of the investigators’ taking notes.

The transcriptions of the audio recordings were coded by three investigators (CN, MtB, and BV) independently, using the methods described by Ose [18]. A conventional content analysis was used to analyse the data, meaning that coding categories were derived directly from the text [19]. The resulting codes were combined into one coding system and categorised by one researcher (BV). The categorization was checked by the other two coding investigators (CN and MtB). The emerging themes are discussed and supported by quotations.

## 3 Results

### 3.1 Database study

The results of the database study can be found in Table 1. In the study period there were 3938 admissions to our ward for 3122 unique patients. A total of 29,993 lab orders including 261,859 individual clinical chemistry tests were ordered. The median length of hospitalization was 4.1 days (interquartile range 1.8–8.3). The mean number of laboratory test orders was 5.7 during the first week of admission, which is well above the Dutch Society of Internal Medicine's recommendation to order lab no more than twice per week. The repeat rate for lab results within normal ranges differs per test, but is generally high, with sodium, potassium, and bicarbonate having the highest rates, at over 80%.

Of the 923 admissions via the emergency room who had a C-reactive protein (CRP) test repeated during admission, 548 patients had (59%) repeat CRP tests performed within 24 h, the recommended minimum testing interval [16]. In 87.8% of repeat CRPs, no effect on patient management, defined as a change in antibiotic therapy, was seen.

Combinations of laboratory tests were common: sodium and potassium were combined in 95% of cases, alanine transaminase (ALT) and aspartate transaminase (AST) in 97%, lipase and amylase in 74%. Procalcitonin, on the other hand, was ordered in combination with CRP in only 8% of CRP test orders.

### 3.2 Survey

**Fig. 1:**
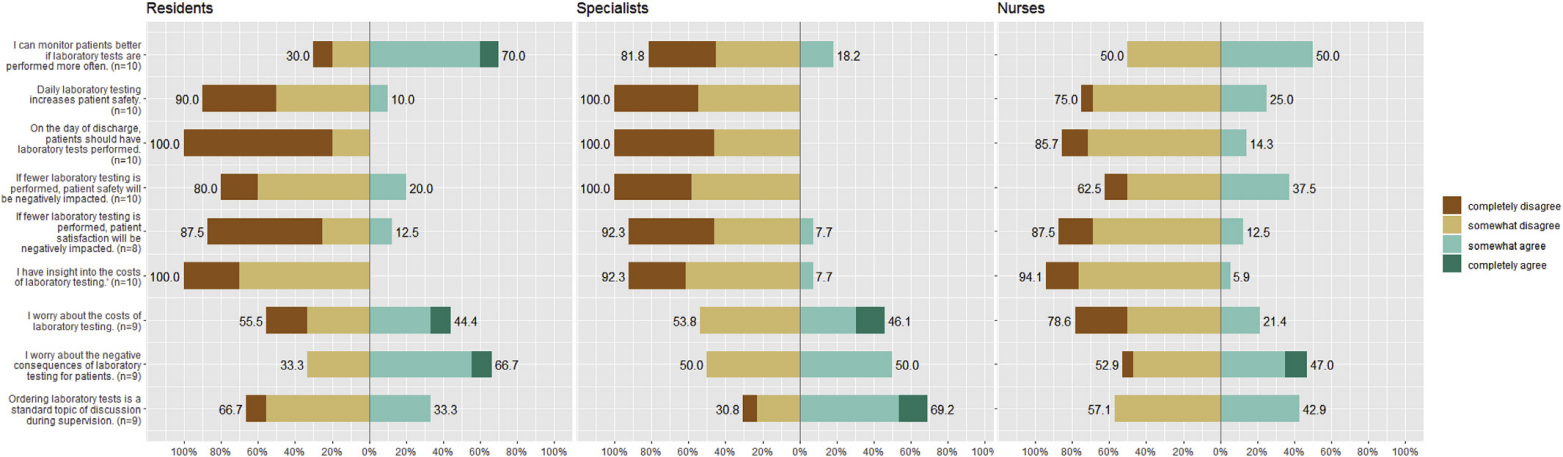
Survey results.

Response rates for the survey were 59% (13/22) for specialists, 14% (10/71) for residents, and 85% (17/20) for nurses. The survey results are presented in Table 2 and Fig. 1. On average, respondents underestimated the weekly number of laboratory orders, with nurses' estimates being the most accurate. The majority of respondents (78%) believed the frequency of lab ordering is too high. Also, the majority of respondents (78%) considered the responsibility for ordering lab tests to lie primarily with the residents.

Regarding the benefits and harms of laboratory testing, 52% of respondents were concerned about the negative consequences of overutilization of laboratory testing for patients. 36% were concerned about the costs of laboratory testing, while only 5% claimed to have insight into the costs. The residents' and specialists' answers were very similar for all but two items. First, residents were more likely than specialists to agree with the statement that frequent laboratory testing helps them to monitor their patients' condition (70% vs. 18%). Second, residents were less likely than specialists to agree with the statement that laboratory testing is discussed during supervision (33% vs. 69%).

### 3.3 Focus groups

Three focus group discussions were held: the first two with residents (R) and the third with specialists (S), comprising 15 participants in total. The causes and solutions of overuse of laboratory testing discussed were divided into three broad categories by the researchers: personal factors, organizational factors and technical factors and further categorised in sub-topics (Table 3).

**Table 3:** Summary of focus group results.

	Causes	Solutions
Personal factors	• lack of knowledge regarding laboratory testing • overtesting being accepted in the context of the residents' learning curve • insecurity of the ordering physician • lack of awareness • lab testing being considered trivial	• creating awareness • reflecting on consequences • conferring with peers
Organizational factors	• specialists not providing feedback to residents • time constraints • lack of education	• more supervision by specialists • feedback on the amounts of testing • feedback and training by clinical pathologists • education
Technical factors	• ease of laboratory testing • not being able to cancel orders	• pop-ups • automated lock-outs

#### 3.3.1 Causes: personal factors

Several personal factors were said to affect inappropriate test ordering. Participants mentioned a lack of knowledge on laboratory testing in general. As one respondent put it: *“How much work [laboratory testing] is, how much it costs, how much normal results can fluctuate, things like that, I think we know very little about that. At least I don't.”* (R8) Residents also noted that improving laboratory ordering is a learning process, in the sense that their lab ordering skills had improved over time during their residency. *“I do think about it more than before. Now, I try to consider whether I really need to order everything.”* (R4) The specialists confirmed this, stating that a certain amount of inappropriate testing is acceptable in order to accommodate the residents' learning process. One specialist used a metaphor to make his point: *“If you let me run the ward, things will go faster and better. And I accept that we don't. Because we want to teach … My child's first time on a bike on the road is a hazard for scratches on other cars. But otherwise they never learn how to ride a bike.” (S1*).

Participants also said often more tests were being ordered than strictly necessary due to the ordering physician's insecurity. As one supervisor said: *“Lab testing is often only done for the doctor's peace of mind.” (S2*).

The personal factor that was being discussed the most was a lack of awareness about test ordering. *“I think that it is something you say very easily: oh, let's do some labs tomorrow. And you may not be aware that, when it's the afternoon, tomorrow is only twelve hours away.”* (R9) Another admitted *“I don't give it much thought really. It's not completely unthinkingly, but to say that it is completely thought through, no.”* (R6).

Many participants expressed that they consider overutilization to be a relatively unimportant issue. *“Nothing really can go wrong,”* said one participant (R9), and another considered it *“too trivial”* (R8).

At the same time, most participants did express concern about the consequences of inappropriate testing. Most participants were primarily concerned about the consequences for patients, such as *“It is a burden to have a venipuncture every day” (S4)* and *“We're making them anemic.” (S3)*, while the financial consequences were considered to be unimportant. As some participants said: *“The costs aren't that high.” (S4)* and *“Adding a CRP test costs, I think, only a few euros.” (R6)*. Yet, these consequences do not appear to be an important factor in the actual decision-making on test ordering. As one participant stated: *“I think we don't have a lot of problems with it ourselves. It's more a problem financially and a burden on patients and nurses. But I don't think it affects me personally if too many lab tests are done.” (R8*).

#### 3.3.2 Causes: organizational factors

Participants discussed multiple organizational factors that cause inappropriate test ordering. The most important factor, according to residents, was a lack of adequate supervision and feedback from their supervisors on their ordering behaviour and culture of not questioning which tests a supervisor suggests. For instance: *“Very little attention is paid to lab ordering. Also when I'm being supervised. That is my opinion at least.”* (R7) or *“Well, often the supervisor just says to run some tests, and I just accept that without question.”* (R3).

Supervisors agreed that they generally did not discuss test ordering with the residents. As one supervisor stated: *“The things I check on a detailed level are the things that make patients live longer or not. But not what lab tests to do tomorrow.”* (S1) Time constraints were mentioned as an important cause of the supervisors not discussing lab ordering with the residents. As one supervisor said: *“Yes, when the supervisor comes running past, then the resident doesn't bring up lab tests, I think.” (S2)* Not all residents expressed a desire to be supervised on laboratory testing. As one resident put it: *“Let me just figure some things out for myself.”* (R3).

Both residents and supervisors said there was hardly any formal education on laboratory testing: *“You are totally dependent on your direct supervisor for what you learn about it.”* (R3) Residents also indicated that there is variability between hospitals. One resident recalled: *“I recently ordered a lipase, but then the gastroenterologist called me and said: in this hospital, we always combine it with an amylase.”* (R3).

#### 3.3.3 Causes: technical factors

The technical factor that was mentioned most often by participants was the ease of laboratory testing, both with regards to the ordering as to the actual blood drawing, especially in patients with intravascular access readily available. *“Central venous access or an intra-arterial line does lower the barrier,”* one participant (R1) mentioned. The digital order form was perceived to be an important facilitator, because of the lack of any barrier for adding more tests. For instance residents stated that when *“Checking boxes on the lab form, I often go, let's do this one too, and that one … "* (R1) and that.

*“When you're ordering lab tests, it is easy to just order some more tests.”* (R6).

Several of the residents also mentioned the inconvenient process of cancelling laboratory orders which involves calling the laboratory. “*Because you order a test, and then, later, you think, oh silly goose, let's cancel it, but then you have to call. And something else comes up and you forget to call*.” (R2).

#### 3.3.4 Solutions: personal factors

The possible solutions that were discussed parallelled the causes. With respect to personal factors, most participants argued that creating awareness was essential. As one participant said: *“You have to think about every single laboratory test ordered.”* (S2). Another said: *“It is all about doing things consciously. And that consciousness has to be created.”* (S3). Reflecting on the consequences of inappropriate testing was thought to be an effective way to increase awareness: *“I noticed a great difference when, for my research, I had to do venipunctures myself. Then you notice how much work it is, and what you're doing to a patient, especially if you have to do a second blood draw.”* (R8).

Another way to increase awareness that was mentioned was to confer with peers. For instance: *“When you're unsure, you could ask your fellow resident. We do that sometimes, but not as often as we could.”* (R2). Another resident suggested a weekly evaluation: *“On the ward, there are two or three residents. So, at the end of the week, you ask each other: how many lab tests did you order and looking back, was it all necessary?”* (R6).

#### 3.3.5 Solutions: organizational factors

With regards to organizational factors several possible solutions were discussed. In the focus groups with residents, the role of the supervisor was discussed at length. Many residents believed more supervision by the specialists could improve lab ordering behaviour. As one resident said: *“Becoming more aware of the problem only happens when someone points it out to you.”* (R1) There were some concerns about the manner of feedback: *“It has to be practicable. I mean – I would become rather grouchy if it was hammered home every day during supervision.”* (R3) and *“I don't want to be micro-managed.”* (R9).

Residents generally felt that feedback on the volume and appropriateness of testing performed would stimulate them to be more critical, functioning as *“a wake-up call”* (R5). Both residents and specialists would appreciate feedback and training by clinical pathologists. For example:

*“I believe that would be incredibly useful. Just more background information and more awareness.” (R8*).

*“Yes, I think it would be very good if you [clinical pathologists] would be more critical about when new lab tests are needed and what to order.” (S4*).

Residents and specialists had different opinions on the effectiveness of education in reducing inappropriate testing. Residents were generally favourable. As one put it: *“I think education is extremely useful. Just more background knowledge and more awareness.”* (R8). Most specialists were not as convinced that education would lead to changes in lab ordering behaviour: *“I have the feeling that, in that respect, education really has zero effect.”* (S2).

#### 3.3.6 Solutions: technical factors

Technical solutions that were suggested included pop-up messages and automated lockouts. Pop-up messages were thought by some to be potentially effective. As one specialist said: *“You might start to think: oh, maybe it’s not necessary.”* (S3). However, most participants were skeptical: *“I don't think that you will still give [pop-up messages] much thought if you see them every day.”* (R4). Another concern was that pop-up messages would be *“rather annoying”* (R5).

Regarding lockouts, residents and specialists had different opinions. Most residents were skeptical, saying for instance that *“they only increase the work load.”* (R8), whereas specialists were mostly in favour: *“With everything that's been tried, also in other hospitals, if you want this, it only works top down. And apparently, it has to be done with lockouts, because the other measures don't work.”* (S4). Still, specialists did not think that lockouts alone would be a good strategy. *“The downside of only applying restrictions is that you lose the opportunity to actually teach the residents.”* (S1).

## 4 Discussion

While the prevalence of laboratory test overutilization is known to be high [2], this study also showed that clinicians often underestimate the actual extent of overutilization. We found health care providers to be ambivalent about the problem. On the one hand, most respondents indicated that they consider overutilization to be a relevant problem, both in terms of patient safety and financially. On the other hand, however, most focus group participants admitted to considering the problem relatively trivial in comparison to other aspects of medical care. Even when physicians profess to find laboratory test overutilization important, in daily practice they don't give laboratory test ordering much thought, citing time constraints.

The most important causes of overutilization, as identified through focus groups, were personal factors, such as a lack of awareness of overutilization and knowledge about appropriate testing, and feelings of insecurity. The causes of overutilization identified by our focus groups are similar to causes found in other studies, such as a lack of understanding of costs, diagnostic uncertainty, and fear of not having the lab results when requested by supervisors [[20], [21], [22]]. Fear of malpractice suits, which has also been found to be a driver of overtesting, could not be identified as such in this study [23].

With regards to potential solutions, opinions differed on what would be the most effective interventions to reduce overtesting. Most residents said they would appreciate more education and direct feedback on appropriate laboratory test utilization, whereas most specialists were more in favour of technical solutions, such as lockouts.

Most survey respondents believed that the residents were most responsible for laboratory ordering, and therefore, it may be argued that residents should be targeted, and that the intervention most favoured by them, more education, may be the most applicable. As many focus group participants stated, clinical pathologists can play a vital role here.

On the other hand, residents frequently rotate between departments, so interventions that only target residents may not produce lasting effects. Therefore, specialists need to be involved and a multifaceted approach that addresses the needs of both residents and specialists may be warranted. It may be effective to combine educational measures with automated lockouts, which are relatively easy and inexpensive to implement, and have the bonus of providing feedback at the moment of test ordering [9].

One of the strengths of this study is that we used multiple research modalities, including a database study, a survey and focus groups, to look at the topic from different angles. Also, we included nurses, residents and specialists in this study, which provides insight from most health care workers involved.

This study has several limitations. First of all, we could not give exact estimates of the amount of inappropriate lab ordering. Because of the large number of hospital admissions, it was not feasible to perform a chart review on all individual admissions to determine whether a test order was actually appropriate or not, but instead we looked at aggregates of the laboratory test results only.

Also, the question of what constitutes inappropriateness does not have a clear cut answer [24]. Not all of the measures we evaluated are covered by guidelines, and even when guidelines apply, they do not always provide unambiguous answers. For instance, in the full text of the Netherlands Association of Internal Medicine's recommendation to order laboratory tests no more than twice a week, the phrase “unless indicated” is added, which begs the question [13].

Secondly, this is a single centre study conducted in a large academic hospital. The results may therefore not be generalisable to other settings, as overutilization of laboratory tests has been shown to be more common in teaching hospitals than in general hospitals [25].

Thirdly, the response rate of residents in our survey was low. This may have affected the outcomes, as residents who are more concerned about the harms of overutilization could be more likely to fill out the questionnaire.

In conclusion, laboratory test overutilization is a common problem with many causes. The most important causes we found were a lack of awareness and knowledge. Interventions to reduce overutilization that were most favoured in our study were education and automated lock-outs.

This study can be used as a template for others to identify local practices and causes of inappropriate laboratory test utilization, which can help identify which interventions are most likely to be successful.

## Provenance and peer review

Not commissioned, externally peer reviewed.

## Ethical approval

This study was deemed to be exempt from review by the Medical Research Ethics Committee of the University Medical Centre Utrecht. The reference number for their judgment is 17/180.

## Sources of funding

No funding was received for this research.

## Author contribution

Bram Vrijsen: study design, data collection, data analysis and interpretation, writing the paper.

Christiana Naaktgeboren: study design, data collection, data analysis and interpretation, writing the paper.

Laura Vos: study design, data analysis, writing the paper.

Wouter van Solinge: study design.

Karin Kaasjager: study design.

Maarten ten Berg: study design, data collection, data analysis and interpretation, writing the paper.

## Consent

N/A.

## Trial registry number

1. Name of the registry: Researchregistry.

2. Unique Identifying number or registration ID: researchregistry5221.

3. Hyperlink to the registration (must be publicly accessible): https://www.researchregistry.com/browse-the-registry#home/?view_2_page=1&view_2_search=5221.

## Guarantor

Bram Vrijsen.

## Declaration of competing interest

None of the authors have any actual or potential conflict of interest to disclose pertaining to this study.
